# Determining Genetic Causal Variants Through Multivariate Regression Using Mixture Model Penalty

**DOI:** 10.3389/fgene.2018.00077

**Published:** 2018-03-05

**Authors:** V. S. Sundar, Chun-Chieh Fan, Dominic Holland, Anders M. Dale

**Affiliations:** ^1^Center for Multimodal Imaging and Genetics, University of California, San Diego, La Jolla, CA, United States; ^2^Department of Radiology, University of California, San Diego, La Jolla, CA, United States; ^3^Department of Cognitive Sciences, University of California, San Diego, La Jolla, CA, United States; ^4^Department of Neuroscience, University of California, San Diego, La Jolla, CA, United States; ^5^Department of Psychiatry, University of California, San Diego, La Jolla, CA, United States

**Keywords:** effect sizes, SNP discovery, optimization, mixture model, fine-mapping

## Abstract

With the availability of high-throughput sequencing data, identification of genetic causal variants accurately requires the efficient incorporation of function annotation data into the optimization routine. This motivates the need for development of novel methods for genome wide association studies with special focus on fine-mapping capabilities. A penalty function method that is simple to implement and capable of integrating functional annotation information into the estimation procedure, is proposed in this work. The idea is to use the prior distribution of the effect sizes explicitly as a penalty function. The estimates obtained are shown to be better correlated with the true effect sizes (in comparison with a few existing techniques). An increase in the positive and negative predictive value is demonstrated using Hapgen2 simulated data.

## 1. Introduction

Detection and estimation of the genetic causal variants associated with a particular phenotypic trait is one of the most challenging problems in modern day statistical genetics. Mathematical techniques are formulated with primary focus on fine-mapping studies, phenotype prediction, and heritability estimation (Servin and Stephens, 2007; Lee et al., 2009; Gaffney et al., 2012; Maller et al., 2012; Valdar et al., 2012; Zuber et al., 2012; de los Campos et al., 2013; International Multiple Sclerosis Genetics Consortium et al., 2013; Zhou et al., 2013; Mahajan et al., 2014; Pickrell, 2014; Spain and Barrett, 2015; Schweiger et al., 2016). Algorithms that integrate functional annotation data into the estimation procedure (Schork et al., 2013; Zhou et al., 2013; Kichaev et al., 2014; Zablocki et al., 2014; Vilhjálmsson et al., 2015) are being continually developed with the understanding that Linkage Disequilibrium (LD) and polygenicity reduces the likelihood of the identified genetic variant being biologically causal (Visscher et al., 2012). The resulting procedures have better fine-mapping and effect size estimation capabilities (Kichaev et al., 2014; Kichaev and Pasaniuc, 2015).

While fine-mapping studies focuses on detecting causal variants, regression, or Bayesian optimization methods integrate these fine-mapping results into the estimation procedure to accurately determine the effect sizes. Currently, fine-mapping studies either use summary statistics or raw genotype data to arrive at quantitative assessment of causal nature of the SNPs. For example, CAVIAR (Hormozdiari et al., 2014, 2015), PAINTOR (Kichaev et al., 2014), and RiVIERA (Li and Kellis, 2016) use summary statistics, and DAP (Wen et al., 2016), CavMeN (Brown et al., 2017) use the raw genotype data. End results of these analyses, typically probability of the SNP being causal, are used in target gene identification studies.

With the understanding that GWAS significant SNPs harbor more than one causal variant, few researchers have attempted to utilize multivariate methods to detect additional association signals (Newcombe et al., 2016; Ning et al., 2017) from summary statistics. Newcombe et al. (2016) utilized the correlation structure of the variants from the reference panel to develop a Bayesian regression framework that accounts for various models with respect to the number of causal SNPs per region. Ning et al. (2017) used the covariance structure between the variants, and between the variant and phenotype vector to obtain LASSO results for a series of λ's (regularization parameter). These studies demonstrate the improvement achieved through additional analysis on already identified potential causal SNPs. The current line of work follows a similar strategy wherein prior information regarding the causal nature of the SNPs (in terms of *p*-values or posterior probabilities) are used to localize additional causal variants (using raw genotype data) that might have been gone undetected due to their small effect size, lower posterior probability, or possibly due to small sample size.

Frequently, a two-mixture model, one each to represent the causal and null SNPs, is used to model the effect size distribution obtained using Genome Wide Association Studies (GWAS) (Meuwissen et al., 2001; Wray et al., 2007; Bukszár et al., 2009; Logsdon et al., 2010; Park et al., 2010, 2011; Yang et al., 2010, 2011; Guan and Stephens, 2011; Habier et al., 2011; Xu et al., 2011; Speed et al., 2012; Zhou et al., 2013; Holland et al., 2016). Accurate identification of causal and null SNPs helps in understanding the underlying biological pathway regulating a disease (Sun et al., 2006; Yoo et al., 2009). Integrating functional annotation data into the estimation procedure is one way of improving the identifiability of causal SNPs (Schork et al., 2013; Kichaev et al., 2014; Zablocki et al., 2014; Kichaev and Pasaniuc, 2015; Vilhjálmsson et al., 2015).

The currently available methods for variable selection and estimating the effect sizes can be broadly categorized into Bayes' theorem based or penalty function based. Bayesian methods proceed by assigning a prior probability density function (pdf) to effect sizes and use either maximum likelihood estimation method or Markov Chain Monte Carlo (MCMC) simulations to determine the posterior effect sizes (effect sizes conditioned on the measured phenotypic data). The various methods that fall under this category differ in the specification of the prior pdf (Meuwissen et al., 2001; Habier et al., 2011; Zhou et al., 2013). Some of them are the Bayesian alphabet models (BayesA, BayesB, BayesC, BayesCπ, BayesR, etc.), and Bayesian Sparse Linear Mixture Model (BSLMM). Regression methods, on the other hand, aim to minimize an objective function with a penalty term, which is chosen to impart sparse characteristics to the effect size estimates. For example, the least angle absolute shrinkage operator (LASSO) uses a *L*_1_ penalty (Tibshirani, 1996), and the Ridge Regression (RR) uses the *L*_2_ penalty (Hoerl and Kennard, 1970). Methods that are a combination of either Bayesian and regression methods (Bayesian LASSO; Park and Casella, 2008; Li et al., 2010) or two regression based methods (Elastic Net; Zou and Hastie, 2005) have also been developed.

While in the Bayesian methods, the prior probabilities aid in variable selection, the shrinkage constraints does the equivalent job in regression based methods. Both the Bayesian and regression methods are geared toward accurate identification of the causal variants and phenotypic prediction. A review of the currently available methods can be found in Zhou et al. (2013) and de los Campos et al. (2013). The Bayesian methods though are mostly independent of tuning parameters, suffer from practical applicability to large datasets (in terms of efficient effect size estimation).

In this work, we formulate a simple and efficient optimization routine which combines the flexibility of Bayesian methods and simplicity of penalty function methods into a single framework. The idea is to use the prior pdf of the effect sizes explicitly as a penalty function. The motivation of the paper is to introduce the method, provide details regarding the implementation of the procedure, and demonstrate its various capabilities. At this stage, theoretically, we do not claim superiority over existing methods developed for effect size estimation and phenotype prediction.

## 2. Problem statement

Considering *N* individuals, *n* genetic markers, and a linear model; the *N* × 1 phenotype vector ***y*** is related to *N* × *n* genotype matrix **X** through the *n* × 1 vector of effect sizes β as (Meuwissen et al., 2001):

(1) y=Xβ+ε

Here ε is the vector of noise terms modeled as *N*(0, Σ_ε_). Elements of **X** are typically coded as 0, 1, or 2 (prior to normalization). We aspire to select the causal variants and determine their effect sizes, $\hat{\beta }$ such that ε = (**X** β − ***y***)^*T*^ (**X**β − ***y***) is minimun. Due to the correlated and sparse nature of the SNPs, the univariate results often end up being erroneous estimates (Kim et al., 2009; de los Campos et al., 2013; Zhou et al., 2013).

This resulted in the evolution of multivariate methods for determining the causal variants (see, for example, Hoerl and Kennard, 1970; Tibshirani, 1996; Zou and Hastie, 2005; Kim et al., 2009; de los Campos et al., 2013; Zhou et al., 2013).

## 3. Methods

### 3.1. Regression methods

The Elastic Net (EN) (Zou and Hastie, 2005) provides the most generalized representation of the commonly used objective functions in penalty methods, and is given as:

(2) $$\begin{array}{l} F_{EN}={\left(X\beta -y\right)}^{T}(X\beta -y)+\lambda \sum_{j=1}^{n}[\frac{1}{2}(1-\alpha )\beta_{j}^{2}+\alpha |\beta_{j}|];\\ \;\lambda >0;\;0<\alpha \leq 1 \end{array}$$

In the above expression, α = 1 corresponds to the LASSO (Tibshirani, 1996), and α = 0 results in the Ridge regression (RR) (Hoerl and Kennard, 1970). There exists several algorithms that minimizes the above objective function while efficiently determining the regularization parameters (Tibshirani, 1996; Fu, 1998; Efron et al., 2004; Zou and Hastie, 2005; Park and Casella, 2008; Wu and Lange, 2008). A variant of the LASSO, termed group LASSO (Meier et al., 2008) employs multiple regularization parameters to different groups of SNPs.

### 3.2. Bayesian methods

Modeling β as a mixture pdf, β ~ π_1_*p*_1_(β) + (1 − π_1_)*p*_0_(β) (Habier et al., 2011; de los Campos et al., 2013; Zhou et al., 2013), with *p*_1_(β) denoting pdf of the causal SNPs and *p*_0_(β) denoting the pdf of the null SNPs, the posterior pdf of β is given as

(3) p(β|y)=Kp(y|β)p(β)

with $K^{-1}=\int_{-\infty }^{\infty }p(y|\beta )p(\beta )d\beta$. The estimate of the effect sizes is determined as 〈β|***y***〉, where 〈•〉 denotes the mathematical expectation operator. Irrespective of the distribution of β, the likelihood function, *p*(***y***|β) can be shown to be Normal with mean **X**β, and variance Σ_ε_ (Robert, 2004). Existing variants of the Bayesian methods could be obtained by changing the pdfs *p*_1_(β) and *p*_0_(β) (de los Campos et al., 2013; Zhou et al., 2013). Supplementary Material provides information on the equivalence between Bayesian and regression based methods for a few priors.

### 3.3. Mixture model penalty method

We design a penalty function that intuitively accomplishes shrinking the regression coefficients while incorporating any prior information about the causal nature of the SNPs. The motivation for this penalty function stems from the understanding that the effect sizes can be realistically represented using a multimodal pdf (Meuwissen et al., 2001; Bukszár et al., 2009; Logsdon et al., 2010; Guan and Stephens, 2011; Habier et al., 2011; Yang et al., 2011; Zhou et al., 2013; Holland et al., 2016) and functional annotations help in classifying the SNPs as either being causal or not (Schork et al., 2013). In our formulation, the main error minimizing term is the negative log-likelihood function, and a mixture prior cost function imparts the necessary sparsity to the effect size estimates. Several researchers in the genetics community have used the Spike and Slab pdf (Ishwaran and Rao, 2005) as prior pdf of effect sizes (de los Campos et al., 2013; Zhou et al., 2013). However, using this pdf explicitly as a penalty function has not been attempted in genetic association studies. This also sidesteps the computationally expensive Markov Chain Monte Carlo (MCMC) method used for obtaining posterior effect size estimates.

The likelihood function, *p*(***y***|β) ~ *N*(**X**β, Σ_ε_) is expressed as

(4) $$p(y|\beta )=\frac{1}{{\left(2\pi \right)}^{n/2}|\Sigma_{\varepsilon }{|}^{1/2}}e^{-\frac{1}{2}{\left(y-X\beta \right)}^{T}\Sigma_{\varepsilon }^{-1}(y-X\beta )}$$

We construct a cost function, *C* that has the ability to capture the causal nature of SNPs:

(5) $$C=\sum_{j=1}^{n}c_{j}({\hat{\beta }}_{j})$$

where the cost associated with the *j*th SNP is given as

(6) $$c_{j}(\beta_{j})=-\text{log}[{\tilde{\pi }}_{1j}p_{1j}(\beta_{j})+(1-{\tilde{\pi }}_{1j})p_{0j}(\beta_{j})]$$

Here ${\tilde{\pi }}_{1}={\left[{\tilde{\pi }}_{11},{\tilde{\pi }}_{12},\cdots \;,{\tilde{\pi }}_{1n}\right]}^{T}$ is the *n* × 1 vector of non-null prior probabilities associated with the functional annotation of the SNPs. That is, if the *j*th SNP is highly likely to be causal, then a higher value (say 0.5) is specified to that SNP. *p*_1*j*_(•) and *p*_0*j*_(•) denote the pdf of causal and null SNPs, respectively. The cost function, thus, acts as a medium to incorporate the enrichment details of individual SNPs. Typically, we use a normal pdf, $\phi_{1j}\left(•;0;{\tilde{\sigma }}_{1j}\right)$, to model the causal effects. Note that ${\tilde{\pi }}_{1}$ denotes the assumed prior probability and π_1_ denotes the true unknown probability. Denoting by *L* the negative log-likelihood function, the function to be minimized is written as

(7) F=L+C

with

(8) $$L=-\text{log}[{\left(2\pi \right)}^{-n/2}|{\tilde{\Sigma }}_{\varepsilon }{|}^{-1/2}]+\frac{1}{2}{\left(y-X\beta \right)}^{T}{\tilde{\Sigma }}_{\varepsilon }^{-1}(y-X\beta )$$

The nonlinear conjugate gradient method (NCG) (Hestenes and Stiefel, 1952; Fletcher and Reeves, 1964; Polak and Ribiere, 1969; Shewchuk, 1994; Dai and Yuan, 1999; Hager and Zhang, 2006) with Newton-Raphson line search algorithm is used to minimize *F*. A step-wise implementation of the optimization procedure is given in Supplementary Material. We show that when the NCG method is used for optimization, the evaluation of whole *n* × *n* Hessian matrix can be avoided. This significantly reduces the computational cost whilst not compromising the accuracy of the solution (Equation S12).

#### 3.3.1. Remarks

1. The cost function shown in Equation (6) is conceptually similar to the penalty function proposed by Ročková and George (2016). The authors, using a mixture Laplace pdf, estimate the prior probability of the effects using a coordinate-wise optimization routine. We, however, specify different prior probabilities for each variant, so as to incorporate any information on LD or functional annotations. Furthermore, our motivation to use a mixture model stems from our understanding of the genetic architecture of the human genome.
2. The likelihood ratio and the cost function are weighed equally, so that the minimum error solution is sparse. Unequal weights can be specified, say higher for *L* if it is known that the genetic architecture is highly polygenic, and low if only a few genetic causal variants influence the phenotype under consideration.
3. Variants of the proposed method could be obtained by changing the pdfs used in constructing the mixture model—for example, Laplace or non-local pdfs (Johnson and Rossell, 2010) could be used instead of two normal pdfs. These however, are minor modifications, and our main contribution lies in proposing an explicit mixture model pdf as a penalty function.
4. Instead of considering cost associate with individual SNPs, the SNPs can be clustered through specification of suitable correlation. This could possibly capture the underlying LD information. However, this requires incorporation of LD metrics such as *r*^2^ into covariance structure of the clustered SNPs. Such studies have not been pursued in this present work, and will likely be a part of future efforts.
5. Higher prior probabilities can be specified to cluster of SNPs in a given LD block that is envisioned to contain the causal signal. SNPs not in this LD block may be provided lower or zero prior probability.
6. SNPs that belong to certain functional annotation category have higher likelihood of being causal. Hence, SNPs in these regions are deemed to be enriched, i.e., have higher probability of influencing a particular phenotype. Typically SNPs tagging regulatory and coding regions are considered to be enriched in comparison with introns and intergenic SNPs (Schork et al., 2013). SNPs in the MHC region can be considered to be enriched when studying immune related diseases (Ellinghaus et al., 2016).
7. Using existing packages such as CAVIAR, DAP, PAINTOR, RiVIERA, and S-LDSC (Finucane et al., 2015), one could obtain a quantitative assessment of the causal nature of individual SNPs. These results can be directly used as prior probabilities (${\tilde{\pi }}_{1}$) in the proposed optimization routine. Probabilities could also be based on GWAS *p*-values. However, these values tend to alter with increase in power.
8. As mentioned earlier, for each regression based method, there exists a Bayesian equivalent. In the Bayesian methods, assuming a prior pdf, samples are drawn from the posterior distribution using MCMC. The proposed method avoids sampling from the prior and posterior pdf of the effect sizes by specifying the prior information explicitly as a penalty function. This distinguishes the method from the Bayesian LASSO and BSLMM.
9. Fine-mapping methods typically require data from dense genotyping arrays, which are further imputed using reference panels, such as 1,000 Genomes (1000 Genomes Project Consortium et al., 2012). The mixture-model method, on the other hand, uses whole genome wide data to locate the causal signal. In this aspect, genotype data preferred for fine-mapping studies, may be unsuitable for the proposed method.

### 3.4. Simulation studies

Hapgen2 (Su et al., 2011) and 1,000 Genomes (1000 Genomes Project Consortium et al., 2012) is used for simulating realistic genotypes for an European population of size 100,000 considering all the 22 chromosomes (80378054 SNPs). True effect sizes are simulated based on the understanding that a proportion of the SNPs are causal with effect sizes distributed as *N*(0, 1).

#### 3.4.1. Whole genome analysis

For whole genome analysis, due to computational issues, the analysis can be carried out using SNP windows such that no two potential causal SNPs in LD are separated (Berisa and Pickrell, 2016). In this work, we consider SNPs associated with individual chromosomes in each sliding window. For each chromosome, the first 20,000 SNPs with minor allele frequency >0.01 are considered in the analysis, resulting in a total of 440,000 SNPs. The number of causal variants are taken to be 50% of the SNPs in the functional annotation category—Exon, 3′UTR, 5′UTR. This gives rise to 4,233 causal SNPs—approximately 1% of the total SNPs considered. Thus, SNPs belonging to these categories are considered to be enriched, i.e., have higher likelihood of being causal. Three different genotype matrices and three different true effect sizes are considered for the analysis, resulting in a total of 18 cases for estimating the normalized mean squared error (NMSE). The phenotype is simulated using Equation (1) with a heritability of 0.5. Specifying prior probability to individual SNPs requires functional annotation information for the genotyped SNPs. Assuming such significant information is unavailable, we initially specify equal ${\tilde{\pi }}_{1}$ and ${\tilde{\sigma }}_{1}$ values for all the SNPs, i.e., ${\tilde{\pi }}_{1j}={\tilde{\pi }}_{1},{\tilde{\sigma }}_{1j}=1,\forall j$—Mixture Model with Constant Priors (MM-CP). An estimate of Σ_ε_ for determining the likelihood function is obtained by assuming a heritability of 0.0227 per SNP window. For comparison, results are obtained using the Regularized Pseudo Inverse (RegPI)—analytical solution with ${\tilde{\pi }}_{1}=\pi_{1}$ and no mixture model (Supplementary Material), LASSO, and univariate regression method. The enrichment factors used in simulating the data are specified as prior probabilities in the optimization routine, and the resulting estimates are denoted as Mixture-Model with Enriched Priors (MM-EP), that is, ${\tilde{\pi }}_{1}=\pi_{1}$. The RegPI method and MM-EP methods differ only in the procedure followed to obtain the effect size estimates, and in principle, are equivalent. While the RegPI method provides a closed form solution for the effect sizes, the MM-EP method utilizes an optimization algorithm to achieve the same goal. As mentioned in section 3.3.1, results from a prior analysis (typically fine-mapping studies) could be used to improve the detection and estimation capabilities of the method. We use end results of DAP as prior probabilities and the resulting estimates are denoted as MM-DAP. It is to be noted that the MM-DAP method utilizes the genotype information twice, once for estimating the prior probability of causality (DAP), and once in our optimization routine (for effect size estimation and detection of additional causal variants). However, the context in which the information is used slightly differs. An adaptive/iterative method for estimating the causal probabilities could avoid this. We are working toward achieving this. Matlab implementation of the proposed method is included along with this paper. The implementation has provision for gradual increment or decrement of ${\tilde{\pi }}_{1}$ and σ_0_ values.

## 4. Results

**Figure 1:**
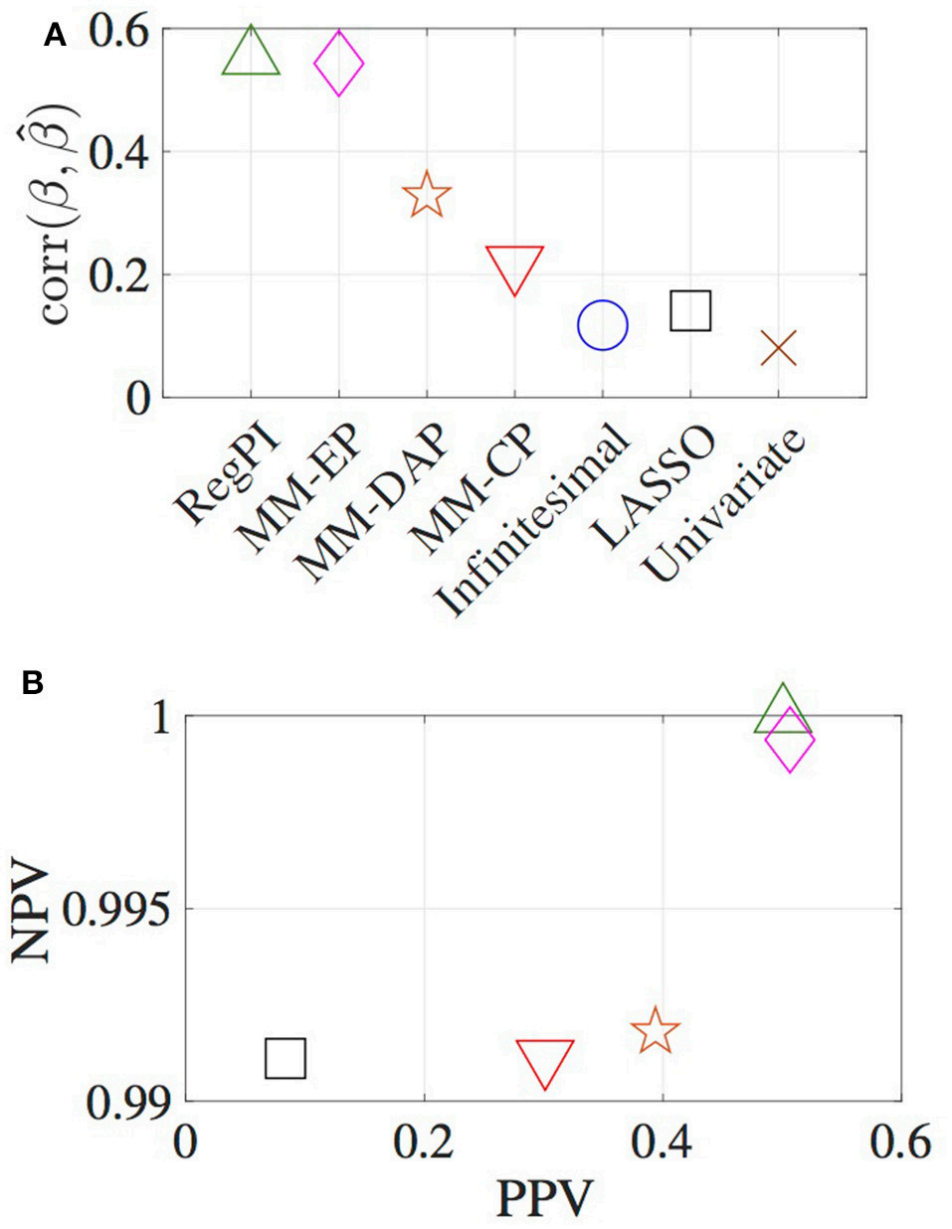
Estimates obtained using various methods. **(A)** correlation between the estimated and true effect sizes, **(B)** PPV and NPV. RegPI: Regularized pseudo inverse (green triangle); MM-EP: Mixture model with enriched priors (magenta diamond); MM-DAP: Mixture model with DAP priors (orange star); MM-CP: Mixture model with constant priors (red inverted triangle); Infinitesimal: Normal prior (no mixture) (blue circle); LASSO (black square); Univariate (brown cross).

The correlation between the true and estimated effect sizes, the percentage of positive and negative predictive values (PPV, NPV) are used to measure the accuracy of different methods (Figure 1). PPV is defined as the ratio of number of true variants identified to the total number of variants identified. Similarly, NPV is defined as the ratio of number of true null SNPs identified to the total number of null SNPs identified. For the RegPI and MM-EP methods, ${\tilde{\pi }}_{1}=\pi_{1}$, hence these methods provide an upper bound for both the effect size correlation, PPV and NPV. This can be considered as a case where complete genetic architecture of the effect size distribution is known. For the MM-CP method, ${\tilde{\pi }}_{1j}=0.01\forall j$, and the null pdf is taken to be Laplacian. Specifying ${\tilde{\pi }}_{1j}=1$ is the special case of the infinitesimal model, where all the SNPs are assumed to be causal with Normal effect size distribution. The MM-DAP method used DAP results as prior probabilities for the genetic variants. This constitutes partial knowledge about the distribution of causal SNPs in the genome.

The MM-CP, Infinitesimal, LASSO, and Univariate methods do not use functional annotation information. Thus any improvement in the effect size estimates obtained using MM-CP method, in comparison with the other three, is deemed significant. Though a sparse structure is imposed on the penalty function in the MM-CP method, the method essentially does not incorporate any enrichment factors. A slight improvement in the effect size correlation can be observed in Figure 1. The figure illustrates the advantage of the proposed formulation in terms of locating the causal variants accurately. The positive and negative predictive value for the Infinitesimal and Univariate methods are not shown in the figure. In obtaining the LASSO estimates, initially, a two-fold cross validation has been carried out for SNP window 1 (i.e., chromosome 1), resulting in λ = 0.508. NMSE estimates are obtained for a grid of values between 0.45 and 0.70 for all the other chromosomes, and the estimates corresponding to the minimum NMSE value is reported in Figure 1. The MM-DAP estimates lie between the MM-CP and MM-EP estimates, as the prior probabilities are based on a previous analysis which identifies few significant SNPs.

**Figure 2:**
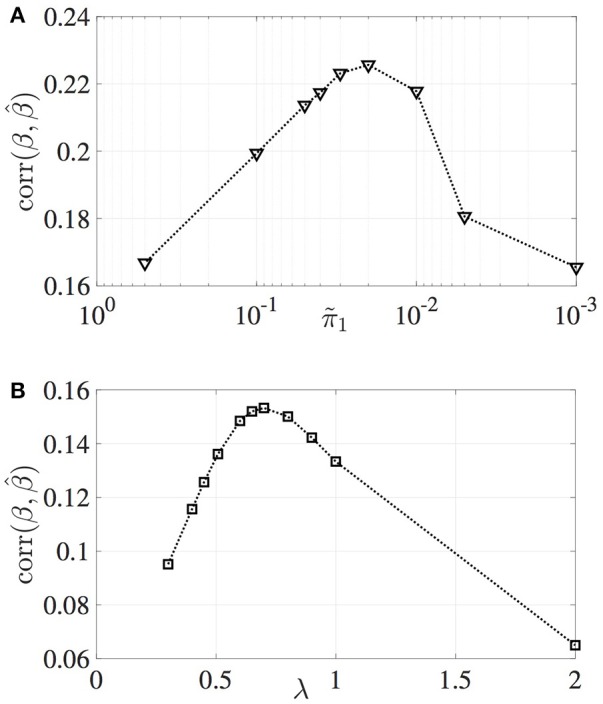
Variation in the correlation between estimated and true effect sizes. **(A)** MM-CP, **(B)** LASSO.

**Figure 3:**
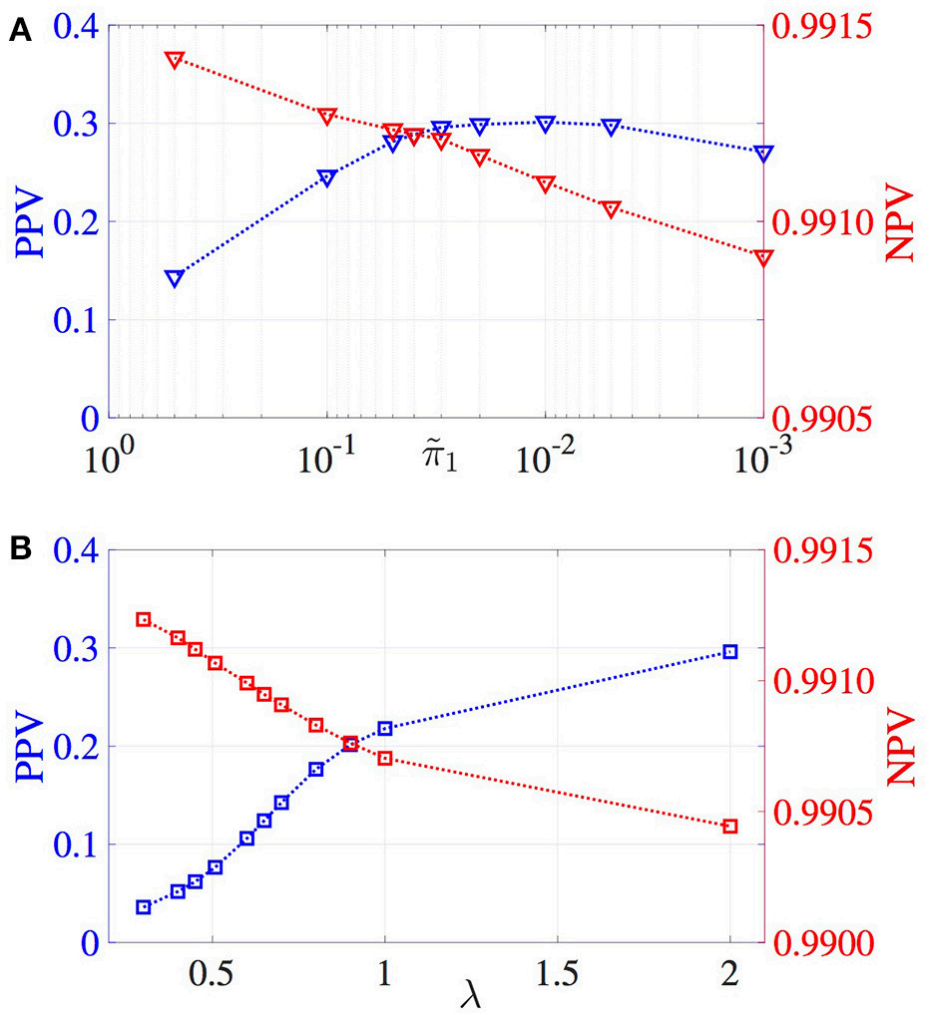
Variation of PPV and NPV values. **(A)** MM-CP, **(B)** LASSO.

The correlation between estimated and true effect sizes, PPV and NPV values have been obtained for a grid of ${\tilde{\pi }}_{1}$ values and plotted in Figures 2, 3, respectively. Similar study is carried out for LASSO (with respect to the regularization parameter λ). For the MM-CP method, the x-axis is plotted in the reverse direction so that moving along the x-axis toward right implies increase in sparseness (consistent with the x-axis of LASSO).

## 5. Discussion

From Figures 2, 3, it can be observed that the MM-CP and LASSO estimates follow a similar trend with increase in sparseness. Enforcing a sparse structure with arbitrary prior probabilities for the effect sizes result in estimates that are better at localizing the genetic causal variants. A good understanding of the distribution of the SNPs across the genome helps in incorporating the functional annotations, thereby further improving the effect size estimates—RegPI and MM-EP methods. A sensible choice of the enrichment factors require prior knowledge about the phenotype under study. For example, SNPs in the MHC region are shown to have a larger impact on Ankylosing Spondylitis than the non MHC SNPs (Ellinghaus et al., 2016). In this case, one could provide a higher π_1_ value for the SNPs in the MHC region (differential enrichment). The MM-DAP results are obtained using this strategy, i.e. use prior information to improve the performance of the estimation procedure.

The correlation plotted in Figure 2 reflects the accuracy of the estimation procedures, that is, how close the estimated effect size is to the true effect size (which is unknown). Here ${\tilde{\pi }}_{1}=1$ implies that all the SNPs (regression coefficients) contribute to the phenotype **y**. This leads to the underestimation of the effect sizes due to the distribution of the true signal among several SNPs. The sparse representation, say ${\tilde{\pi }}_{1}=10^{-2}$ on the other hand has the advantage of distributing the total signal among few selected non-zero SNPs. Depending on the selected SNPs, the correlation between true and estimated SNPs may vary. For the infinitesimal case, though all the causal SNPs have been identified, will result in low correlation value, because the effect sizes of these causal variants have been underestimated. The same phenomenon can be observed when the infinitesimal model (${\tilde{\pi }}_{1}=1$) is compared with LASSO in Figure 1.

It is straightforward to note that the amount of shrinkage achieved depends on the characteristics of the penalty function used. Using the mixture model pdf explicitly as a penalty function places a probabilistic sparse constraint on the effect sizes, as opposed to the distance based constraints used typical in penalty function based method. Specifying a sparse structure without any knowledge about the underlying genetic architecture (i.e., specifying an arbitrary π_1_) is shown to equip the optimization routine with better fine-mapping capabilities, and result in estimates that are at least as good as the LASSO and Univariate methods.

The non-linear conjugate gradient method is used to solve the optimization problem efficiently by harnessing the structure of the objective function's Hessian matrix (Supplementary Material). Thus, the method in its current form can be applied to whole genome analysis without any difficulty. Application of the method to specialized chip sequenced data, say the Immunochip or Oncochip, requires a careful approach in specifying the ${\tilde{\pi }}_{1}$ values to various SNPs. We are working toward developing methodologies to automatically determine the probability of SNP association, and SNP correlation with in the optimization framework. Alternately, end results from existing fine-mapping studies such as DAP or PAINTOR can be used as prior probabilities. Therefore, the method needs to be viewed as an efficient optimization algorithm capable of integrating functional annotation data (if available). Interpretation of the framework as a means to incorporate functional annotation and LD information, while at the same time achieving good variable selection and effect size estimation capabilities are some of the features we believe are important to the genetics community.

## Author contributions

VS: Wrote manuscript, performed analyses, contributed to study design, interpretation of results, and critical revision of manuscript; C-CF and DH: Contributed to data preparation, interpretation of results, and critical revision of manuscript; AD: Performed analyses, contributed to study design, interpretation of results, and critical revision of manuscript.

### Conflict of interest statement

The authors declare that the research was conducted in the absence of any commercial or financial relationships that could be construed as a potential conflict of interest.

## Supplementary material

The Matlab code along with toy example problem can be downloaded freely from the author's website: https://sites.google.com/site/sundarvelkur/code-with-sample-data.

## References

[B1] 1000 Genomes Project Consortium Abecasis G. R. Auton A. Brooks L. D. DePristo M. A. Durbin R. M.. (2012). An integrated map of genetic variation from 1,092 human genomes. Nature 491, 56–65. 10.1038/nature11632 23128226 PMC3498066

[B2] Berisa T. Pickrell J. K. (2016). Approximately independent linkage disequilibrium blocks in human populations. Bioinformatics 32, 283–285. 10.1093/bioinformatics/btv546 26395773 PMC4731402

[B3] Brown A. A. Viñuela A. Delaneau O. Spector T. D. Small K. S. Dermitzakis E. T. (2017). Predicting causal variants affecting expression by using whole-genome sequencing and rna-seq from multiple human tissues. Nat. Genet. 49:1747. 10.1038/ng.3979 29058714

[B4] Bukszár J. McClay J. L. van den Oord E. J. (2009). Estimating the posterior probability that genome-wide association findings are true or false. Bioinformatics 25, 1807–1813. 10.1093/bioinformatics/btp305 19420056 PMC2705227

[B5] Dai Y.-H. Yuan Y. (1999). A nonlinear conjugate gradient method with a strong global convergence property. SIAM J. Optimiz. 10, 177–182.

[B6] de Los Campos G. Hickey J. M. Pong-Wong R. Daetwyler H. D. Calus M. P. (2013). Whole-genome regression and prediction methods applied to plant and animal breeding. Genetics 193, 327–345. 10.1534/genetics.112.143313 22745228 PMC3567727

[B7] Efron B. Hastie T. Johnstone I. Tibshirani R. (2004). Least angle regression. Ann. Stat. 32, 407–499. 10.1214/009053604000000067

[B8] Ellinghaus D. Jostins L. Spain S. L. Cortes A. Bethune J. Han B.. (2016). Analysis of five chronic inflammatory diseases identifies 27 new associations and highlights disease-specific patterns at shared loci. Nat. Genet. 48, 510–518. 10.1038/ng.3528 26974007 PMC4848113

[B9] Finucane H. K. Bulik-Sullivan B. Gusev A. Trynka G. Reshef Y. Loh P.-R.. (2015). Partitioning heritability by functional annotation using genome-wide association summary statistics. Nat. Genet. 47, 1228–1235. 10.1038/ng.3404 26414678 PMC4626285

[B10] Fletcher R. Reeves C. M. (1964). Function minimization by conjugate gradients. Comput. J. 7, 149–154. 10.1093/comjnl/7.2.149

[B11] Fu W. J. (1998). Penalized regressions: the bridge versus the lasso. J. Comput. Graph. Stat. 7, 397–416.

[B12] Gaffney D. J. Veyrieras J.-B. Degner J. F. Pique-Regi R. Pai A. A. Crawford G. E.. (2012). Dissecting the regulatory architecture of gene expression QTLs. Genome Biol. 13:R7. 10.1186/gb-2012-13-1-r7 22293038 PMC3334587

[B13] Guan Y. Stephens M. (2011). Bayesian variable selection regression for genome-wide association studies and other large-scale problems. Ann. Appl. Stat. 5, 1780–1815. 10.1214/11-AOAS455

[B14] Habier D. Fernando R. L. Kizilkaya K. Garrick D. J. (2011). Extension of the bayesian alphabet for genomic selection. BMC Bioinformatics 12:186. 10.1186/1471-2105-12-186 21605355 PMC3144464

[B15] Hager W. W. Zhang H. (2006). A survey of nonlinear conjugate gradient methods. Pac. J. Optim. 2, 35–58.

[B16] Hestenes M. R. Stiefel E. (1952). Methods of conjugate gradients for solving linear systems. J. Res. Natl. Bureau Stand. 49, 409–436.

[B17] Hoerl A. E. Kennard R. W. (1970). Ridge regression: biased estimation for nonorthogonal problems. Technometrics 12, 55–67.

[B18] Holland D. Wang Y. Thompson W. K. Schork A. Chen C.-H. Lo M.-T.. (2016). Estimating effect sizes and expected replication probabilities from gwas summary statistics. Front. Genet. 7:15. 10.3389/fgene.2016.00015 26909100 PMC4754432

[B19] Hormozdiari F. Kichaev G. Yang W.-Y. Pasaniuc B. Eskin E. (2015). Identification of causal genes for complex traits. Bioinformatics 31, i206–i213. 10.1093/bioinformatics/btv240 26072484 PMC4542778

[B20] Hormozdiari F. Kostem E. Kang E. Y. Pasaniuc B. Eskin E. (2014). Identifying causal variants at loci with multiple signals of association. Genetics 198, 497–508. 10.1534/genetics.114.167908 25104515 PMC4196608

[B21] International Multiple Sclerosis Genetics Consortium (IMSGC) Beecham A. H. Patsopoulos N. A. Xifara D. K. Davis M. F. Kemppinen A.. (2013). Analysis of immune-related loci identifies 48 new susceptibility variants for multiple sclerosis. Nat. Genet. 45, 1353–1360. 10.1038/ng.2770 24076602 PMC3832895

[B22] Ishwaran H. Rao J. S. (2005). Spike and slab variable selection: frequentist and bayesian strategies. Ann. Stat. 33, 730–773. 10.1214/009053604000001147

[B23] Johnson V. E. Rossell D. (2010). On the use of non-local prior densities in bayesian hypothesis tests. J. R. Stat. Soc. Ser. B 72, 143–170. 10.1111/j.1467-9868.2009.00730.x

[B24] Kichaev G. Pasaniuc B. (2015). Leveraging functional-annotation data in trans-ethnic fine-mapping studies. Am. J. Hum. Genet. 97, 260–271. 10.1016/j.ajhg.2015.06.007 26189819 PMC4573268

[B25] Kichaev G. Yang W.-Y. Lindstrom S. Hormozdiari F. Eskin E. Price A. L.. (2014). Integrating functional data to prioritize causal variants in statistical fine-mapping studies. PLoS Genet. 10:e1004722. 10.1371/journal.pgen.1004722 25357204 PMC4214605

[B26] Kim S. Sohn K.-A. Xing E. P. (2009). A multivariate regression approach to association analysis of a quantitative trait network. Bioinformatics 25, i204–i212. 10.1093/bioinformatics/btp218 19477989 PMC2687972

[B27] Lee S.-I. Dudley A. M. Drubin D. Silver P. A. Krogan N. J. Pe'er D.. (2009). Learning a prior on regulatory potential from eQTL data. PLoS Genet. 5:e1000358. 10.1371/journal.pgen.1000358 19180192 PMC2627940

[B28] Li J. Das K. Fu G. Li R. Wu R. (2010). The bayesian lasso for genome-wide association studies. Bioinformatics 27, 516–523. 10.1093/bioinformatics/btq688 21156729 PMC3105480

[B29] Li Y. Kellis M. (2016). RiVIERA-MT: a bayesian model to infer risk variants in related traits using summary statistics and functional genomic annotations. bioRxiv 059345. 10.1101/059345

[B30] Logsdon B. A. Hoffman G. E. Mezey J. G. (2010). A variational bayes algorithm for fast and accurate multiple locus genome-wide association analysis. BMC Bioinformatics 11:58. 10.1186/1471-2105-11-58 20105321 PMC2824680

[B31] Mahajan A. Go M. J. Zhang W. Below J. E. Gaulton K. J. Ferreira T.. (2014). Genome-wide trans-ancestry meta-analysis provides insight into the genetic architecture of type 2 diabetes susceptibility. Nat. Genet. 46, 234–244. 10.1038/ng.2897 24509480 PMC3969612

[B32] Maller J. B. McVean G. Byrnes J. Vukcevic D. Palin K. Su Z.. (2012). Bayesian refinement of association signals for 14 loci in 3 common diseases. Nat. Genet. 44, 1294–1301. 10.1038/ng.2435 23104008 PMC3791416

[B33] Meier L. Van De Geer S. Bühlmann P. (2008). The group lasso for logistic regression. J. R. Stat. Soc. Ser. B 70, 53–71. 10.1111/j.1467-9868.2007.00627.x

[B34] Meuwissen T. H. Hayes B. J. Goddard M. E. (2001). Prediction of total genetic value using genome-wide dense marker maps. Genetics 157, 1819–1829. 11290733 10.1093/genetics/157.4.1819 PMC1461589

[B35] Newcombe P. J. Conti D. V. Richardson S. (2016). JAM: a scalable bayesian framework for joint analysis of marginal snp effects. Genet. Epidemiol. 40, 188–201. 10.1002/gepi.21953 27027514 PMC4817278

[B36] Ning Z. Lee Y. Joshi P. K. Wilson J. F. Pawitan Y. Shen X. (2017). A selection operator for summary association statistics reveals allelic heterogeneity of complex traits. Am. J. Hum. Genet. 101, 903–912. 10.1016/j.ajhg.2017.09.027 29198721 PMC5812891

[B37] Park J.-H. Gail M. H. Weinberg C. R. Carroll R. J. Chung C. C. Wang Z.. (2011). Distribution of allele frequencies and effect sizes and their interrelationships for common genetic susceptibility variants. Proc. Natl. Acad. Sci. U.S.A. 108, 18026–18031. 10.1073/pnas.1114759108 22003128 PMC3207674

[B38] Park J.-H. Wacholder S. Gail M. H. Peters U. Jacobs K. B. Chanock S. J.. (2010). Estimation of effect size distribution from genome-wide association studies and implications for future discoveries. Nat. Genet. 42, 570–575. 10.1038/ng.610 20562874 PMC4615599

[B39] Park T. Casella G. (2008). The bayesian lasso. J. Am. Stat. Assoc. 103, 681–686. 10.1198/016214508000000337

[B40] Pickrell J. K. (2014). Joint analysis of functional genomic data and genome-wide association studies of 18 human traits. Am. J. Hum. Genet. 94, 559–573. 10.1016/j.ajhg.2014.03.004 24702953 PMC3980523

[B41] Polak E. Ribiere G. (1969). Note sur la convergence de méthodes de directions conjuguées. Revue Française d'informatique et de Recherche Opérationnelle. Série Rouge 3, 35–43.

[B42] Robert C. P. (2004). Monte Carlo Methods. Wiley Online Library.

[B43] Ročková V. George E. I. (2016). The spike-and-slab lasso. J. Am. Stat. Assoc. 10.1080/01621459.2016.1260469

[B44] Schork A. J. Thompson W. K. Pham P. Torkamani A. Roddey J. C. Sullivan P. F. (2013). All SNPs are not created equal: genome-wide association studies reveal a consistent pattern of enrichment among functionally annotated SNPs. PLoS Genet. 9:e1003449 10.1371/journal.pgen.1003449 23637621 PMC3636284

[B45] Schweiger R. Kaufman S. Laaksonen R. Kleber M. E. März W. Eskin E.. (2016). Fast and accurate construction of confidence intervals for heritability. Am. J. Hum. Genet. 98, 1181–1192. 10.1016/j.ajhg.2016.04.016 27259052 PMC4908190

[B46] Servin B. Stephens M. (2007). Imputation-based analysis of association studies: candidate regions and quantitative traits. PLoS Genet. 3:e114. 10.1371/journal.pgen.0030114 17676998 PMC1934390

[B47] Shewchuk J. R. (1994). An Introduction to the Conjugate Gradient Method Without the Agonizing Pain. Carnegie Mellon University.

[B48] Spain S. L. Barrett J. C. (2015). Strategies for fine-mapping complex traits. Hum. Mol. Genet. 24, R111–R119. 10.1093/hmg/ddv260 26157023 PMC4572002

[B49] Speed D. Hemani G. Johnson M. R. Balding D. J. (2012). Improved heritability estimation from genome-wide SNPs. Am. J. Hum. Genet. 91, 1011–1021. 10.1016/j.ajhg.2012.10.010 23217325 PMC3516604

[B50] Su Z. Marchini J. Donnelly P. (2011). Hapgen2: simulation of multiple disease SNPs. Bioinformatics 27, 2304–2305. 10.1093/bioinformatics/btr341 21653516 PMC3150040

[B51] Sun L. Craiu R. V. Paterson A. D. Bull S. B. (2006). Stratified false discovery control for large-scale hypothesis testing with application to genome-wide association studies. Genet. Epidemiol. 30, 519–530. 10.1002/gepi.20164 16800000

[B52] Tibshirani R. (1996). Regression shrinkage and selection via the lasso. J. R. Stat. Soc. Ser. B 58, 267–288.

[B53] Valdar W. Sabourin J. Nobel A. Holmes C. C. (2012). Reprioritizing genetic associations in hit regions using lasso-based resample model averaging. Genet. Epidemiol. 36, 451–462. 10.1002/gepi.21639 22549815 PMC3470705

[B54] Vilhjálmsson B. J. Yang J. Finucane H. K. Gusev A. Lindström S. Ripke S.. (2015). Modeling linkage disequilibrium increases accuracy of polygenic risk scores. Am. J. Hum. Genet. 97, 576–592. 10.1016/j.ajhg.2015.09.001 26430803 PMC4596916

[B55] Visscher P. M. Brown M. A. McCarthy M. I. Yang J. (2012). Five years of GWAS discovery. Am. J. Hum. Genet. 90, 7–24. 10.1016/j.ajhg.2011.11.029 22243964 PMC3257326

[B56] Wen X. Lee Y. Luca F. Pique-Regi R. (2016). Efficient integrative multi-snp association analysis via deterministic approximation of posteriors. Am. J. Hum. Genet. 98, 1114–1129. 10.1016/j.ajhg.2016.03.029 27236919 PMC4908152

[B57] Wray N. R. Goddard M. E. Visscher P. M. (2007). Prediction of individual genetic risk to disease from genome-wide association studies. Genome Res. 17, 1520–1528. 10.1101/gr.6665407 17785532 PMC1987352

[B58] Wu T. T. Lange K. (2008). Coordinate descent algorithms for lasso penalized regression. Ann. Appl. Stat. 2, 224–244. 10.1214/07-AOAS147 PMC3212875 22081779

[B59] Xu L. Craiu R. V. Sun L. (2011). Bayesian methods to overcome the winner's curse in genetic studies. Ann. Appl. Stat. 5, 201–231. 10.1214/10-AOAS373

[B60] Yang J. Benyamin B. McEvoy B. P. Gordon S. Henders A. K. Nyholt D. R.. (2010). Common snps explain a large proportion of the heritability for human height. Nat. Genet. 42, 565–569. 10.1038/ng.608 20562875 PMC3232052

[B61] Yang J. Weedon M. N. Purcell S. Lettre G. Estrada K. Willer C. J.. (2011). Genomic inflation factors under polygenic inheritance. Eur. J. Hum. Genet. 19, 807–812. 10.1038/ejhg.2011.39 21407268 PMC3137506

[B62] Yoo Y. J. Pinnaduwage D. Waggott D. Bull S. B. Sun L. (2009). Genome-wide association analyses of north american rheumatoid arthritis consortium and framingham heart study data utilizing genome-wide linkage results. BMC Proc. 3:S103. 10.1186/1753-6561-3-S7-S103 20017967 PMC2795874

[B63] Zablocki R. W. Schork A. J. Levine R. A. Andreassen O. A. Dale A. M. Thompson W. K. (2014). Covariate-modulated local false discovery rate for genome-wide association studies. Bioinformatics 30, 2098–2104. 10.1093/bioinformatics/btu145 24711653 PMC4103587

[B64] Zhou X. Carbonetto P. Stephens M. (2013). Polygenic modeling with bayesian sparse linear mixed models. PLoS Genet. 9:e1003264. 10.1371/journal.pgen.1003264 23408905 PMC3567190

[B65] Zou H. Hastie T. (2005). Regularization and variable selection via the elastic net. J. R. Stat. Soc. Ser. B 67, 301–320. 10.1111/j.1467-9868.2005.00503.x

[B66] Zuber V. Silva A. P. D. Strimmer K. (2012). A novel algorithm for simultaneous SNP selection in high-dimensional genome-wide association studies. BMC Bioinformatics 13:284. 10.1186/1471-2105-13-284 23113980 PMC3558454

