# Supplementary material for: Determining Genetic Causal Variants Through Multivariate Regression Using Mixture Model Penalty
Source: Front Genet. 2018 Mar 5;9:77. doi: 10.3389/fgene.2018.00077 (PMC5844985; doi:10.3389/fgene.2018.00077)
Supplement: Supplementary file 1 [file DataSheet1.PDF]

Supplementary Material: Determining genetic causal variants through multivariate regression using mixture model penalty

1 EQUIVALENCE OF RIDGE REGRESSION AND NORMAL PRIOR

This section derives the equivalence between the Ridge Regression and Bayesian normal prior. From Bayes' theorem, we have

$$p(\boldsymbol{\beta}|\boldsymbol{y}) = \frac{p(\boldsymbol{\beta}, \boldsymbol{y})}{p(\boldsymbol{y})}$$

If the prior is assumed to be Gaussian, we can derive an exact expression for the joint probability density function (pdf) of the effect sizes and phenotype vector:

$$p(\boldsymbol{\beta}, \boldsymbol{y}) = N\left(\begin{bmatrix}\mu_{\boldsymbol{\beta}} \\ \mu_{\boldsymbol{Y}}\end{bmatrix}, \begin{bmatrix}\Sigma_{\boldsymbol{\beta\beta}} & \Sigma_{\boldsymbol{\beta Y}} \\ \Sigma_{\boldsymbol{Y\beta}} & \Sigma_{\boldsymbol{YY}}\end{bmatrix}\right)$$

with the variance of the phenotype, and cross covariance of the effect sizes and the phenotye vector given as $\Sigma_{\boldsymbol{YY}} = \mathbf{X}\Sigma_{\boldsymbol{\beta\beta}}\mathbf{X}^t + \Sigma_\varepsilon$ and $\Sigma_{\boldsymbol{\beta Y}} = \Sigma_{\boldsymbol{\beta\beta}}\mathbf{X}^t$ respectively. The expected value and variance of the posterior effect size estimates are given as:

$$\langle \boldsymbol{\beta} | \boldsymbol{Y} \rangle = \mu_{\boldsymbol{\beta}} - \Sigma_{\boldsymbol{\beta\beta}}\Sigma_{\boldsymbol{YY}}(\boldsymbol{y} - \mu_{\boldsymbol{Y}})$$

$$\text{Var}(\boldsymbol{\beta}|\boldsymbol{Y}) = \Sigma_{\boldsymbol{\beta\beta}} - \Sigma_{\boldsymbol{\beta Y}}\Sigma_{\boldsymbol{YY}}^{-1}\Sigma_{\boldsymbol{Y\beta}}$$

The equivalence between the Bayesian methods and Regression based methods is shown next. Considering $\mu_{\boldsymbol{\beta}} = 0$, the posterior pdf of the effect size estimate can be written as

$$\begin{aligned} p(\boldsymbol{\beta}|\boldsymbol{y}) &= Cp(\boldsymbol{y}|\boldsymbol{\beta})p(\boldsymbol{\beta}) \\ &= C\frac{1}{(2\pi)^{n/2}|\Sigma_\varepsilon|^{1/2}}e^{-\frac{1}{2}(\boldsymbol{y}-\mathbf{X}\boldsymbol{\beta})^t\Sigma_\varepsilon^{-1}(\boldsymbol{y}-\mathbf{X}\boldsymbol{\beta})}\frac{1}{(2\pi)^{n/2}|\Sigma_{\boldsymbol{\beta\beta}}|^{1/2}}e^{-\frac{1}{2}\boldsymbol{\beta}^t\Sigma_{\boldsymbol{\beta\beta}}^{-1}\boldsymbol{\beta}} \\ &= C\frac{1}{(2\pi)^n|\Sigma_\varepsilon|^{1/2}}\frac{1}{|\Sigma_{\boldsymbol{\beta\beta}}|^{1/2}}e^{-\frac{1}{2}\{(\boldsymbol{y}-\mathbf{X}\boldsymbol{\beta})^t\Sigma_\varepsilon^{-1}(\boldsymbol{y}-\mathbf{X}\boldsymbol{\beta})+\boldsymbol{\beta}^t\Sigma_{\boldsymbol{\beta\beta}}^{-1}\boldsymbol{\beta}\}} \end{aligned} \tag{S1}$$

From the above equation it is clear that maximizing the likelihood ratio taking the priors into account, i.e. maximizing $p(\boldsymbol{y}|\boldsymbol{\beta})p(\boldsymbol{\beta})$, is similar to minimizing the expression inside the exponential function, i.e. $(\boldsymbol{y} - \mathbf{X}\boldsymbol{\beta})^t\Sigma_\varepsilon^{-1}(\boldsymbol{y} - \mathbf{X}\boldsymbol{\beta}) + \boldsymbol{\beta}^t\Sigma_{\boldsymbol{\beta\beta}}^{-1}\boldsymbol{\beta}$, which is equivalent to the Ridge Regression. Similarly, if a Laplace pdf is assumed as the prior for each of the effect sizes, $\mathcal{L}(0, \sigma_j), j = 1, 2, \cdots, n$, the expression inside the exponential function can be shown to be equal to $(\boldsymbol{y}-\mathbf{X}\boldsymbol{\beta})^t\Sigma_\varepsilon^{-1}(\boldsymbol{y}-\mathbf{X}\boldsymbol{\beta}) + \sum_{j=1}^n \frac{1}{\sigma_j}|\beta_j|$. This is equivalent to the LASSO method of multivariate regression. A very important point to note is that the methods are equivalent only in terms of the function to be minimized and take entirely different routes in arriving at a solution, and interpreting the tuning parameters.

2 ANALYSIS OF THE COST FUNCTION

Consider the case with only one SNP,

$$\begin{aligned} C &= -\log\Big[\tilde{\pi}_{1j}p_1(\beta) + (1-\tilde{\pi}_1)p_0(\beta)\Big] \\ &\approx -w_1\log[\tilde{\pi}_{1j}p_1(\beta)] - w_2\log[(1-\tilde{\pi}_1)p_0(\beta)] \\ &= w_1\frac{\beta^2}{2\sigma_1^2} + w_2\frac{|\beta|}{\sigma_0} - w_1\log\Big[\frac{\pi_1}{\sqrt{2\pi}}\Big] - w_2\log\Big[\frac{(1-\pi_1)}{2\sigma_0}\Big] \text{ (assuming Gaussian-Laplace mixture)} \end{aligned} \tag{S2}$$

The cost function thus acts a regulator with an additional term containing information on the prior probabilities of the SNPs. The weights w_1 and w_2 arise due to the approximation: $\log(A+B) = \log(A) + \log(B)$, and do not appear in the actual optimization routine. The approximation is introduced to show the similarity of the cost function with the Elastic-Net penalty.

3 IMPLEMENTATION

An expression for the standard deviation of the error term could be obtained from the data as

$$\sigma_\varepsilon = \sqrt{(1-h^2)\frac{1}{N}\sum_{i=1}^{N}(\boldsymbol{y}_i)^2} \tag{S3}$$

Here, we assume the heritability, h^2; typically, $0.2 \leq h^2 \leq 0.6$. The gradient vector and the Hessian matrix for the objective function, $F = L + C$, are obtained as follows:

$$\begin{aligned} \text{gradient, } \nabla \boldsymbol{f} &= \frac{\partial L}{\partial \hat{\boldsymbol{\beta}}} + \frac{\partial C}{\partial \hat{\boldsymbol{\beta}}} \\ \text{Hessian, } \mathbf{H} &= \frac{\partial^2 L}{\partial \hat{\boldsymbol{\beta}}_j \partial \hat{\boldsymbol{\beta}}_k} + \frac{\partial^2 C}{\partial \hat{\boldsymbol{\beta}}^2};\ j,k = 1,2,\cdots,n \end{aligned} \tag{S4}$$

with the gradient and Hessian matrix for the likelihood and cost function given as

$$\begin{aligned} \boldsymbol{g}_L &= \frac{\partial L}{\partial \hat{\boldsymbol{\beta}}} \\ &= \mathbf{X}^T\Sigma_\varepsilon^{-1}(\mathbf{X}\hat{\boldsymbol{\beta}} - \boldsymbol{y}) \end{aligned} \tag{S5}$$

$$\begin{aligned} \mathbf{H}_L &= \frac{\partial^2 L}{\partial \hat{\boldsymbol{\beta}}_j \partial \hat{\boldsymbol{\beta}}_k}; j,k = 1,2,\cdots,n \\ &= \mathbf{X}^T\Sigma_\varepsilon^{-1}\mathbf{X}; \end{aligned} \tag{S6}$$

$$\begin{aligned} \boldsymbol{g}_C &= \frac{\partial C}{\partial \hat{\boldsymbol{\beta}}} \\ &\approx \frac{C(\hat{\boldsymbol{\beta}} + \Delta\boldsymbol{\beta}) - C(\hat{\boldsymbol{\beta}} - \Delta\boldsymbol{\beta})}{2\Delta\boldsymbol{\beta}} \end{aligned} \tag{S7}$$

$$
\begin{aligned}
\mathbf{H}_C &= \frac{\partial^2 C}{\partial \hat{\boldsymbol{\beta}}^2} \\
&\approx \frac{C(\hat{\boldsymbol{\beta}} + \Delta\boldsymbol{\beta}) - 2C(\hat{\boldsymbol{\beta}}) + C(\hat{\boldsymbol{\beta}} - \Delta\boldsymbol{\beta})}{\Delta\boldsymbol{\beta}^2}
\end{aligned}
\tag{S8}
$$

In Eq. (S5), the terms $\partial\hat{\boldsymbol{\beta}}_j$ and $\partial\hat{\boldsymbol{\beta}}_k$ are mentioned explicitly to indicate the fact that the the design matrix, $\mathbf{X}$ models the correlation among the β's. The cost function, on the other hand, models the effect sizes as being statistically independent, thus $\mathbf{H}_C$ is a diagonal matrix.

The nonlinear conjugate gradient method with Newton-Raphson line search algorithm is used to minimize F. The mathematical structure of the Hessian matrix is utilized to efficiently obtain the step size for the Newton's line search routine. Denoting the diagonal elements of $\mathbf{H}_C$ with $\boldsymbol{h}_c$, the step size for the Newton line search is given as

$$
\alpha = -\frac{\nabla \boldsymbol{f}^T \boldsymbol{d}}{\boldsymbol{d}^T \mathbf{H} \boldsymbol{d}} = -\frac{(\boldsymbol{g}_L + \boldsymbol{g}_C)\boldsymbol{d}}{\boldsymbol{d}^T(\mathbf{H}_L + \mathbf{H}_C)\boldsymbol{d}}
\tag{S9}
$$

Using Eq. (S5), we get

$$
\boldsymbol{d}^T \mathbf{H}_L \boldsymbol{d} = \boldsymbol{d}^T(\mathbf{X}^T \Sigma_\varepsilon^{-1} \mathbf{X})\boldsymbol{d} = (\mathbf{X}\boldsymbol{d})^T \Sigma_\varepsilon^{-1}(\mathbf{X}\boldsymbol{d})
\tag{S10}
$$

and

$$
\boldsymbol{d}^T \mathbf{H}_C \boldsymbol{d} = \boldsymbol{d}^T(\boldsymbol{h}_c \circ \boldsymbol{d})
\tag{S11}
$$

Here $\circ$ denotes the element-wise vector product (Hadamard product). Thus the step size, α is now given as

$$
\alpha = -\frac{(\boldsymbol{g}_L + \boldsymbol{g}_C)\boldsymbol{d}}{(\mathbf{X}\boldsymbol{d})^T \Sigma_\varepsilon^{-1}(\mathbf{X}\boldsymbol{d}) + \boldsymbol{d}^T(\boldsymbol{h}_c \circ \boldsymbol{d})}
\tag{S12}
$$

From Eq. (S12) it can be clearly seen that the evaluation of the $n \times n$ Hessian matrix is side-stepped by pre-multiplying the design matrix with the conjugate direction vector. This significantly reduces the computational cost whilst not compromising the accuracy of the solution.

A simpler optimization routine would be to use a steepest descent approach for determining the step size. The update in the effect size estimate for such a routine is given as $\boldsymbol{\beta}_{i+1} = \boldsymbol{\beta}_i + d\boldsymbol{\beta}$ with $d\boldsymbol{\beta} = s\boldsymbol{d}$. The search direction, $\boldsymbol{d}$ and scaling factor, s are given as

$$
\boldsymbol{d} = \frac{\nabla \boldsymbol{f}}{||\nabla \boldsymbol{f}||} \text{ and } s = \frac{-\boldsymbol{d}^T \nabla \boldsymbol{f}}{\boldsymbol{d}^T \mathbf{H} \boldsymbol{d}}
\tag{S13}
$$

respectively. Table S1 provides the analytical expressions for the gradient and Hessian for a few objective functions.

A step-wise implementation of the optimization procedure is given in Algorithm 1. In the algorithm, $\boldsymbol{d}$ denotes the conjugate direction; $\boldsymbol{g}_L$, and $\boldsymbol{g}_C$ denote the gradients of the likelihood and cost functions, respectively; and $\boldsymbol{h}_c$ denotes the diagonal elements of the cost function's Hessian matrix, $\mathbf{H}_C$. The mathematical structure of the Hessian matrix is utilized to efficiently obtain the step size for the Newton's line search routine. Supplementary material 3 provides more details regarding the implementation.

Algorithm 1 Nonlinear conjugate gradient method for association studies

1: Set $i = 1$; Initialize $\boldsymbol{\beta}_i = \boldsymbol{\beta}_0$.
2: Determine $\boldsymbol{r}_0 = \boldsymbol{g}_L + \boldsymbol{g}_C$ at $\boldsymbol{\beta} = \boldsymbol{\beta}_i$ using Eqs. (S5) and (S7).
3: Set $\boldsymbol{d}_0 = \boldsymbol{r}_0$.
4: $\alpha = -\frac{(\boldsymbol{g}_L+\boldsymbol{g}_C)\boldsymbol{d}_0}{(\mathbf{X}\boldsymbol{d}_0)^T\Sigma_\varepsilon^{-1}(\mathbf{X}\boldsymbol{d}_0)+\boldsymbol{d}_0^T(\boldsymbol{h}_c\circ\boldsymbol{d}_0)}$
5: $\boldsymbol{\beta}_{i+1} = \boldsymbol{\beta}_i + \alpha\boldsymbol{d}_0$
6: $\boldsymbol{r}_1 = \boldsymbol{g}_L + \boldsymbol{g}_C$ at $\boldsymbol{\beta} = \boldsymbol{\beta}_{i+1}$.
7: $\gamma = \begin{cases} \frac{\boldsymbol{r}_1^T\boldsymbol{r}_1}{\boldsymbol{r}_0^T\boldsymbol{r}_0} & \text{Fletcher-Reeves} \\ \max\{\frac{\boldsymbol{r}_1^T(\boldsymbol{r}_1-\boldsymbol{r}_0)}{\boldsymbol{r}_0^T\boldsymbol{r}_0}, 0\} & \text{Polak-Ribière-Polyak} \\ \frac{\boldsymbol{r}_1^T(\boldsymbol{r}_1-\boldsymbol{r}_0)}{\boldsymbol{d}_0^T(\boldsymbol{r}_1-\boldsymbol{r}_0)} & \text{Henstenes-Stiefel} \\ \frac{\boldsymbol{r}_1^T\boldsymbol{r}_1}{\boldsymbol{d}_0^T(\boldsymbol{r}_1-\boldsymbol{r}_0)} & \text{Dai-Yun} \end{cases}$
8: $\boldsymbol{d}_1 = \boldsymbol{r}_1 + \gamma\boldsymbol{d}_0$
9: If $\boldsymbol{d}_1^T\boldsymbol{r}_1 < \epsilon$, stop;
Else $i = i + 1$, $\boldsymbol{r}_0 = \boldsymbol{r}_1$, $\boldsymbol{d}_0 = \boldsymbol{d}_1$, Goto Step 4.

4 REGULARIZED PSEUDO INVERSE

The objective function to be minimized without considering a mixture model is given as

$$F = -\log\left[(2\pi)^{-n/2}|\Sigma_\varepsilon|^{-1/2}\right] + \frac{1}{2}(\boldsymbol{y} - \mathbf{X}\hat{\boldsymbol{\beta}})^T\Sigma_\varepsilon^{-1}(\boldsymbol{y} - \mathbf{X}\hat{\boldsymbol{\beta}}) - \sum_{j=1}^{n}\log[\pi_1 p_{1j}(\hat{\beta}_j)] \tag{S14}$$

Conditions of optimality:

$$\frac{\partial F}{\partial \beta_j} = 0, j = 1, 2, \cdots, n$$

Assuming normal distribution for the non-null SNPs, $p_{1j} = \phi(\hat{\beta}_j, 0, \sigma_1)$, an analytical expression for the estimated effect size can be derived as

$$\hat{\boldsymbol{\beta}} = \left[\boldsymbol{\pi}_1^{-1}\boldsymbol{\Sigma}_1^{-1} + \mathbf{X}^T\boldsymbol{\Sigma}_\varepsilon^{-1}\mathbf{X}\right]^{-1}\mathbf{X}^T\boldsymbol{\Sigma}_\varepsilon^{-1}\boldsymbol{y} \tag{S15}$$

5 BAYESIAN METHODS

Modeling $\boldsymbol{\beta}$ as a mixture pdf, $\boldsymbol{\beta} \sim \pi p_{\boldsymbol{\beta}_1}(\boldsymbol{\beta}) + (1-\pi)p_{\boldsymbol{\beta}_0}(\boldsymbol{\beta})$, with $p_{\boldsymbol{\beta}_1}(\boldsymbol{\beta})$ denoting pdf of the causal SNPs and $p_{\boldsymbol{\beta}_0}(\boldsymbol{\beta})$ denoting the pdf of the null SNPs, the posterior pdf of $\boldsymbol{\beta}$ is given as

$$p(\boldsymbol{\beta}|\boldsymbol{y}) = Kp(\boldsymbol{y}|\boldsymbol{\beta})p(\boldsymbol{\beta}) \tag{S16}$$

with $K^{-1} = \int_{-\infty}^{\infty} p(\boldsymbol{y}|\boldsymbol{\beta})p(\boldsymbol{\beta})d\boldsymbol{\beta}$. The estimate of the effect sizes is determined as $\langle\boldsymbol{\beta}|\boldsymbol{y}\rangle$, where $\langle\bullet\rangle$ denotes the mathematical expectation operator. Irrespective of the distribution of $\boldsymbol{\beta}$, the likelihood function, $p(\boldsymbol{y}|\boldsymbol{\beta})$ can be shown to be Normal with mean $\mathbf{X}\boldsymbol{\beta}$, and variance Σ_ε. Existing variants of the Bayesian methods could be obtained by changing the pdfs $p_{\boldsymbol{\beta}_1}(\boldsymbol{\beta})$ and $p_{\boldsymbol{\beta}_0}(\boldsymbol{\beta})$.

Table S1. Gradient and Hessian for various methods

Method	Gradient	Hessian
Ridge Regression	$2\mathbf{X}^T(\mathbf{X}\hat{\boldsymbol{\beta}} - \boldsymbol{y}) + 2\lambda\boldsymbol{\beta}$	$2\mathbf{X}^T\mathbf{X} + 2\lambda\mathbf{I}$
LASSO	$2\mathbf{X}^T(\mathbf{X}\hat{\boldsymbol{\beta}} - \boldsymbol{y}) + \lambda\ \mathrm{sgn}(\boldsymbol{\beta})$	$2\mathbf{X}^T\mathbf{X}$
Elastic Net	$2\mathbf{X}^T(\mathbf{X}\hat{\boldsymbol{\beta}} - \boldsymbol{y}) + \lambda[(1-\alpha)\boldsymbol{\beta} + \alpha\ \mathrm{sgn}(\boldsymbol{\beta})]$	$2\mathbf{X}^T\mathbf{X} + \lambda(1-\alpha)\mathbf{I}$
Mixture penalty	$\mathbf{X}^T\Sigma_\varepsilon^{-1}(\mathbf{X}\hat{\boldsymbol{\beta}} - \boldsymbol{y}) + \frac{C(\hat{\beta}+\Delta\beta)-C(\hat{\beta}-\Delta\beta)}{2\Delta\beta}$	$\mathbf{X}^T\Sigma_\varepsilon^{-1}\mathbf{X} + \frac{C(\hat{\beta}+\Delta\beta)-2C(\hat{\beta})+C(\hat{\beta}-\Delta\beta)}{\Delta\beta^2}$

Table S2. Time taken to obtain the estimates using different methods

Method	Time (Hr)
Regularized Pseudo Inverse	23.15
MM-EP	28.18
MM-CP	27.45
LASSO	94.48
Univariate	7.26

6 UNIVARIATE POSTERIOR PROBABILITY

Considering a prior mixture pdf for the effect sizes, $\beta_j \sim \pi_1 N(0,1) + (1-\pi_1)\delta(\beta_j), j = 1, 2, \cdots, n$, an expression for the univariate posterior distribution for the effect sizes is obtained as

$$p(\beta_j|\boldsymbol{y}) = \frac{p(\boldsymbol{y}|\beta_j)p(\beta_j)}{\int_{-\infty}^{\infty} p(\boldsymbol{y}|\beta_j)p(\beta_j)d\beta_j} \tag{S17}$$

which also can be shown to be a mixture model.

7 UNIVARIATE REGRESSION

If $\beta_j \perp\!\!\!\perp \beta_k, \forall j \neq k$, a univariate linear regression analysis would result in the desired solution (Figure S1):

$$\beta_j = \frac{\sum_{i=1}^{N} y_i X_{ij}}{\sum_{k=1}^{N} X_{kj}^2}, j = 1, 2, \cdots, n \tag{S18}$$

Due to the correlated and sparse nature of the SNPs, the univariate results often end up being erroneous estimates. This resulted in the evolution of multivariate methods for determining the causal variants.

8 SUPPLEMENTARY FIGURES AND TABLES

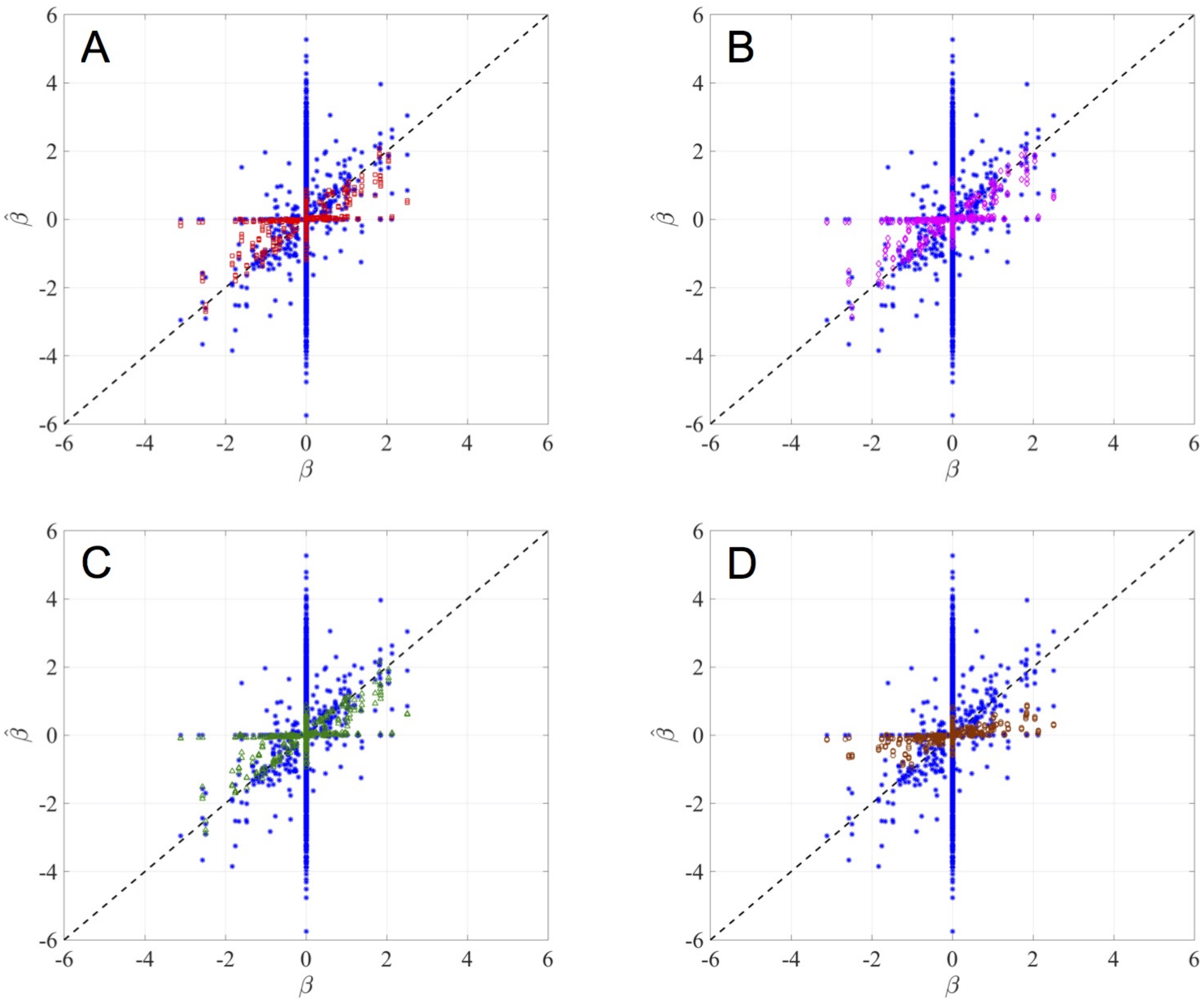

Figure S1: Comparison of the estimates obtained using various $\tilde{\pi}_1$ values and and ordinary least squares (blue stars). (A) $\tilde{\pi}_1 = 10^{-3}$, (B) $\tilde{\pi}_1 = 10^{-2}$, (C) $\tilde{\pi}_1 = 10^{-1}$, (D) $\tilde{\pi}_1 = 1$. Note that using the infinitesimal model (D) distributes the total signal among all the SNP resulting in underestimation of the effect sizes. The sparse model (A-C) improves the performance of the algorithm through effective variable selection, and distributing the total signal among these identified SNPs.

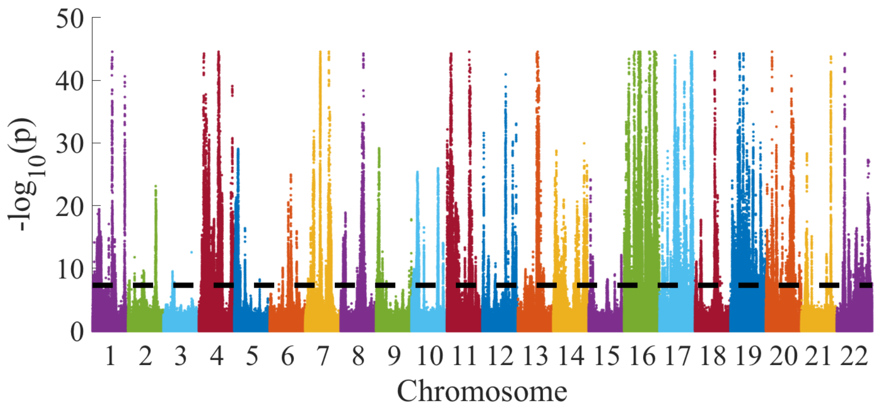

Figure S2: Manhattan plot. Black dashed line denote the genome wide significance threshold.
